# Statin Prescription Patterns and Associations with Subclinical Inflammation

**DOI:** 10.3390/medicina58081096

**Published:** 2022-08-14

**Authors:** Preetham Kadappu, Jitendra Jonnagaddala, Siaw-Teng Liaw, Blake J. Cochran, Kerry-Anne Rye, Kwok Leung Ong

**Affiliations:** 1 School of Medical Sciences, University of New South Wales, Sydney, NSW 2052, Australia; 2 School of Population Health, University of New South Wales, Sydney, NSW 2052, Australia

**Keywords:** atherosclerosis, cardiovascular diseases, hospitalisations, inflammation, prescribing, statins

## Abstract

*Background and Objectives:* Statins have been extensively utilised in atherosclerotic cardiovascular disease (ASCVD) prevention and can inhibit inflammation. However, the association between statin therapy, subclinical inflammation and associated health outcomes is poorly understood in the primary care setting. *Materials and Methods:* Primary care electronic health record (EHR) data from the electronic Practice-Based Research Network (ePBRN) from 2012–2019 was used to assess statin usage and adherence in South-Western Sydney (SWS), Australia. Independent determinants of elevated C-reactive protein (CRP) were determined. The relationship between baseline CRP levels and hospitalisation rates at 12 months was investigated. *Results:* The prevalence of lipid-lowering medications was 14.0% in all adults and 44.6% in the elderly (≥65 years). The prevalence increased from 2012 to 2019 despite a drop in statin use between 2013–2015. A total of 55% of individuals had good adherence (>80%). Hydrophilic statin use and higher intensity statin therapy were associated with elevated CRP levels. However, elevated CRP levels were not associated with all-cause or ASCVD hospitalisations after adjusting for confounders. *Conclusions:* The prevalence and adherence patterns associated with lipid-lowering medications highlighted the elevated ASCVD-related burden in the SWS population, especially when compared with the Australian general population. Patients in SWS may benefit from enhanced screening protocols, targeted health literacy and promotion campaigns, and timely incorporation of evidence into ASCVD clinical guidelines. This study, which used EHR data, did not support the use of CRP as an independent marker of future short-term hospitalisations.

## 1. Introduction

Atherosclerotic cardiovascular disease (ASCVD) is the leading cause of morbidity and mortality globally [1]. Atherosclerosis, which is the deposition of lipids in the intima of blood vessels, is the key process underlying ASCVDs, such as ischaemic heart disease and ischaemic stroke [2].

Statins are the mainstay therapy for ASCVD management, as they lower low-density lipoprotein cholesterol (LDL-C), which reduces incident ASCVD events [3]. Statins also act as anti-inflammatory agents and directly reduce circulating CRP levels, which may contribute to a further reduction in ASCVD event risk [4]. Guidelines recommend aggressive statin use in populations with higher ASCVD risk [5]. However, there is little monitoring of the prescription patterns of, and adherence to, statins in high-risk subpopulations. This study provides valuable feedback to primary practitioners on the adequacy of pharmacological management of ASCVD in their communities.

Furthermore, atherosclerosis has been recognized as an inflammation-driven disease that is characterised by increased subclinical pro-inflammatory cytokine expression and immune cell involvement [6]. C-reactive protein (CRP) has emerged as a key inflammatory marker with great predictive power for ASCVD event rates, but low marginal benefit in risk stratification in existing frameworks [7]. However, results from the CANTOS [8] and LoDoCo [9] trials suggested that inflammation may play a causal role in ASCVD. This suggests that CRP may be useful for monitoring inflammation in patients on anti-inflammatory therapy as a strategy for gauging treatment efficacy and residual ASCVD risk from inflammation.

This present study focused on the use of the real-world electronic health record (EHR) data from the Electronic Practice-Based Research Network (ePBRN), which is a dataset from primary practitioners in South-Western Sydney (SWS). As this region had a significantly lower socioeconomic profile compared with Greater Sydney [10], the study focused on statin-related ASCVD monitoring and outcomes in this demographic. There were three aims of this study. The first aim was to investigate the prevalence patterns and adherence to statin therapy in ePBRN. The second aim was to investigate factors that independently influenced CRP in those taking statins in ePBRN. The third aim was to investigate the relationship between the on-treatment CRP levels with short-term all-cause and ASCVD hospitalisations over a 12-month follow-up period.

## 2. Materials and Methods

### 2.1. Study Setting and Population

**Figure 1:**
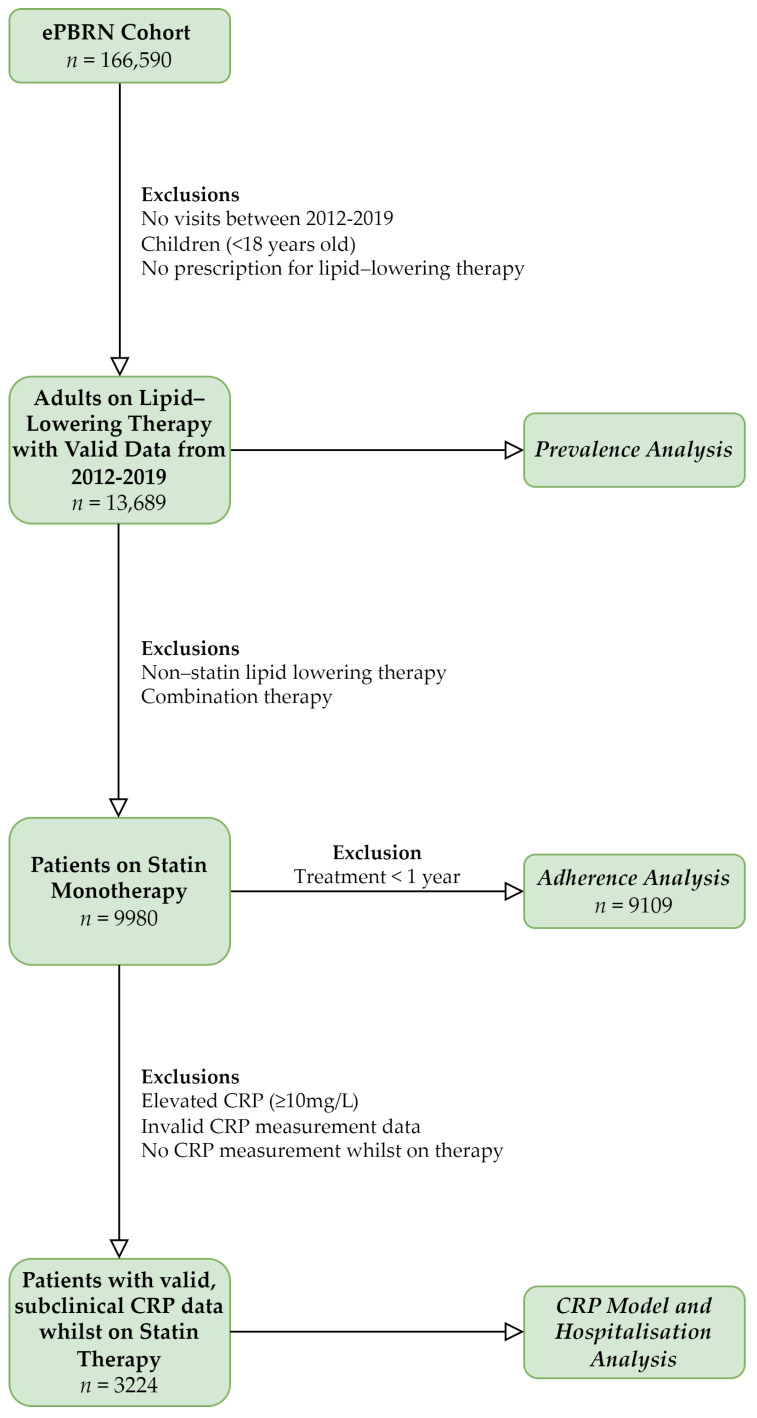
Subject selection in the ePBRN cohort.

The 2012–2019 ePBRN-linked dataset consisted of anonymised, computerised records sourced from a primary care network of general practitioner (GP) clinics located in SWS [11]. These were linked to hospital data using a GRHANITE^TM^ linkage [12]. All adults (age ≥ 18 years) on lipid-lowering therapy were included for prevalence analyses and those on statin monotherapy for over a year were included for adherence analyses. Patients with missing or invalid CRP levels (>10 mg/L) were excluded, as this indicated active, acute inflammation [13]. Different lipid-lowering medications were identified using the World Health Organization (WHO) Anatomical Therapeutic Chemical (ATC) code (Table A1). Only the first CRP measurement was included if multiple CRP measurements were found for a particular patient (Figure 1). The use of data from ePBRN was approved by the UNSW Human Research Ethics Advisory Panel (HC190591; 28 August 2019).

### 2.2. Data Collection

Demographic data, medical history, medication use, lifestyle factors, examination findings and investigation results were obtained as covariate parameters. Key demographic data included age, gender, Aboriginal/Torres Strait Islander (ATSI) status and living location. Chronic medical conditions were grouped by the key system affected according to the International Statistical Classification of Diseases and Related Health Problems 10th Revision (ICD-10) codes. Medication history focused on diabetic, anti-thrombotic and other cardiovascular medications. Examination findings focused on anthropometric measurements, and standard biochemical parameters were included in the study analysis.

In the ePBRN, information for age, gender, Aboriginal/Torres Strait Islander (ATSI) status and living location were captured. The postcodes of self-reported primary residence were categorised as urban and non-urban using the 2015 Modified Monash Model [14]. Smoking status was taken from the most recent recorded data in the ePBRN database. Medication history was assessed through the presence of active prescriptions of diabetic, anti-thrombotic and other cardiovascular medications at the time of the CRP measurement. Statin lipophilicity and intensity of statin therapy were categorised as described elsewhere (Table A2) [15,16]. Relevant biomarkers included full blood counts, clinical biochemistry tests, liver function tests, kidney function tests, coagulation markers, blood glucose and glycosylated haemoglobin (HbA1c), lipid profiles and inflammatory markers. The biomarker data were included if obtained within 2 weeks of the CRP measurement. In the ePBRN, eGFR was obtained directly from the values calculated by pathology clinics, which also used the CKD-EPI equation [17]. The examination findings included blood pressure and body mass index (BMI) measurements. The most recent BMI measurement was used in the analysis. However, blood pressure measurements were used in the analysis only if taken on the same day as the CRP measurement due to the high variability of blood pressure.

In the ePBRN, chronic medical conditions were obtained through the GP and hospital records of diagnoses or procedural interventions. Diabetes was defined as having a recorded diagnosis, fasting blood glucose ≥7.0 mmol/L, HbA1c ≥6.5% or random blood glucose ≥11.1 mmol/L. Hypertension was defined as either having a recorded diagnosis, systolic blood pressure ≥140 mmHg or diastolic blood pressure ≥90 mmHg. Dyslipidaemia was defined as having a prior diagnosis, total cholesterol ≥4.0 mmol/L, LDL-C ≥2.0 mmol/L, high-density lipoprotein cholesterol (HDL-C) <1.0 mmol/L, non-HDL cholesterol ≥2.5 mmol/L, triglycerides ≥2.0 mmol/L or a total-to-HDL-C ratio ≥4 [18].

The medical history of hospital patients was recorded using ICD-10 codes. Medical history data within 12 months following a CRP measurement were extracted and analysed. As there were no data on the reasons for admission, the presence of an acute condition or procedural intervention was used as the reason for admission. Where the reason for the visit was ASCVD or its procedural intervention (such as I20.1 or percutaneous coronary intervention), these hospital admissions were classified as ASCVD events.

### 2.3. Statistical Analysis

Demographics of the cohort, including age, gender, living location and ethnicity were analysed based on the year(s) of the patient visits to the GP and the presence of active prescriptions for lipid-lowering drugs. The *p*-value for the linear time trend was estimated using linear regression.

Statin adherence was calculated using the following equation for patients on statin monotherapy for ≥1 year (*n* = 9109):

Adherence to Medication = (Prescription Coverage)/(Treatment Duration) × 100%

The treatment duration was defined as the number of days between the start of the first prescription and the end of the most recent prescription. The prescription coverage was defined as the number of days where there was an active prescription.

Univariable, logistic regression analysis was performed to assess the association of different variables with elevated CRP levels (defined as a level ≥2 mg/L) [5]. To identify independent determinants of elevated CRP levels, variables with *p* < 0.2 in univariable analysis were entered in a stepwise logistic regression model with backward elimination until all variables were significant (*p* < 0.05). Multicollinearity issues were detected using the variance inflation factor (>5) and issues were resolved by selecting the best fitting model as determined using the *R*^2^ value. The receiver operating characteristic (ROC) analysis was used to assess the model performance.

Cox proportional hazard analysis was used to estimate the hazard ratios for the association of CRP levels with all-cause or ASCVD hospitalisation at the 12-month follow-up. Model 1 adjusted for basic demographic characteristics of age and gender, and model 2 adjusted further for factors that were independently associated with CRP, as determined in the previous analysis. The proportional hazard assumption was checked using Schoenfeld residuals and no violations were found for any model.

All data analysis was performed using IBM SPSS version 24.0 software (SPSS, Inc., Chicago, IL, USA). A two-tailed *p* < 0.05 was considered statistically significant.

## 3. Results

### 3.1. Study Population

The original ePBRN contained 166,590 patients, 13,689 of whom were on lipid-lowering therapy. A total of 9980 patients were on statin monotherapy, 9109 of which had been so for over a year. A total of 3224 patients had valid CRP data and were included in the final analysis (Figure 1).

### 3.2. Statin Usage and Adherence in SWS

**Figure 2:**
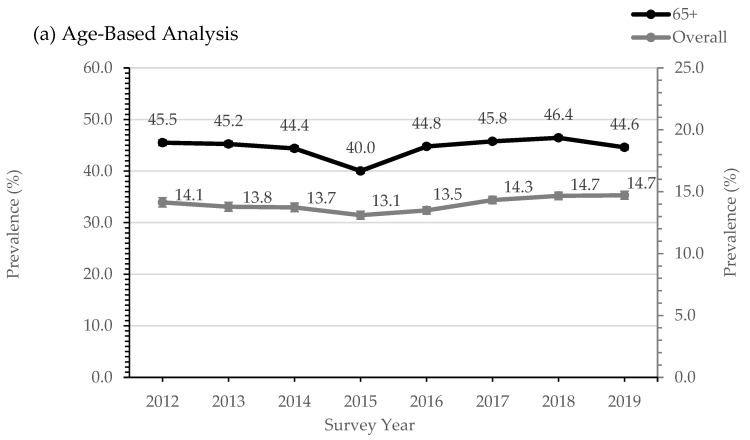

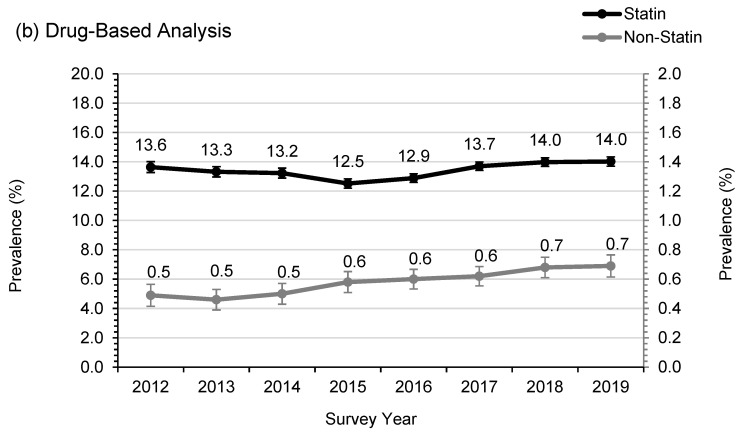
Prevalence of lipid-lowering medications in the ePBRN.

The prevalence of lipid-lowering medications was, on average, 14.0% in adults and 44.6% in those aged ≥65 years. Overall, the prevalence increased from 14.1% in 2012 to 14.7% in 2019 (*p* for time trend = 0.001) despite a drop from 13.8% in 2013 to 13.1% in 2015 (*p* for time trend <0.001), which was led by a decrease in statin use (Figure 2).

Statins comprised over 85% of the lipid-lowering medications prescribed. Two-thirds of subjects were on moderate-intensity statin therapy, 30% were on high-strength statin therapy and <3% were on low-strength statin therapy (Table A3). Men tended to be on higher-strength statin therapy compared with females (Table A4). When examining the cohort of lipid-lowering medication users, the mean age and proportion of urban residents increased from 2012 to 2019 (*p* < 0.05), but there were no significant changes in the gender composition or ATSI status over time (Table A5).

**Figure 3:**
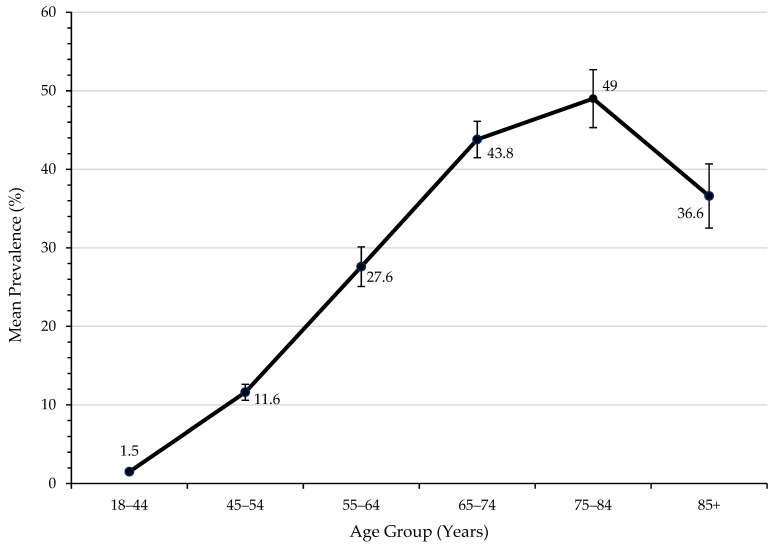
Prevalence of lipid-lowering medication use by age.

Prescription patterns were further examined in subgroups of age, gender and living location (Table A6). Prevalence tended to increase with age; however, it sharply dropped in those aged ≥65 years (Figure 3). Conversely, non-statin lipid-lowering therapy did not differ between age groups (*p* > 0.05) (Table A6).

The prevalence of all lipid-lowering medications increased in both males and females over time (*p* < 0.05), but males had a higher prevalence than females (Table A6). The percentage of urban residents receiving statin therapy increased over time, whereas the percentage of non-urban residents receiving statins declined (*p* < 0.05). However, the percentage of non-statin lipid-lowering therapy grew in both regions over time (*p* < 0.05) (Table A6).

**Figure 4:**
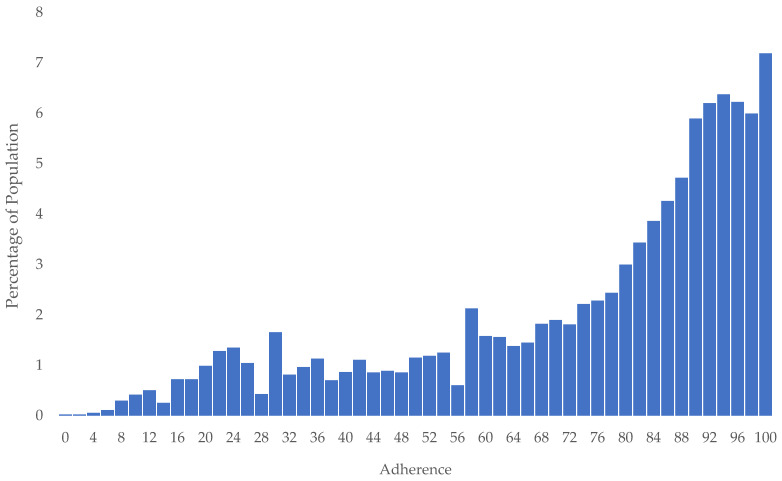
Frequency histogram of statin adherence.

As seen in Figure 4 there was a positive skew toward full adherence. The median adherence was 83% (interquartile range: 60–93%). A total of 55% of the cohort had good adherence (≥80%). From the univariable analysis, older age and urban residence were associated with good adherence, and these were independently associated with good adherence based on the multivariable analysis (*p* < 0.05) (Table A7).

### 3.3. Factors Associated with Elevated CRP Levels

Among the 3224 patients in the ePBRN on statin monotherapy with a valid CRP measurement, the mean age was 67.2 years. Men comprised 47.9% of this cohort and the percentage of those with elevated CRP was 57.1% (Table A8).

**Figure 5:**
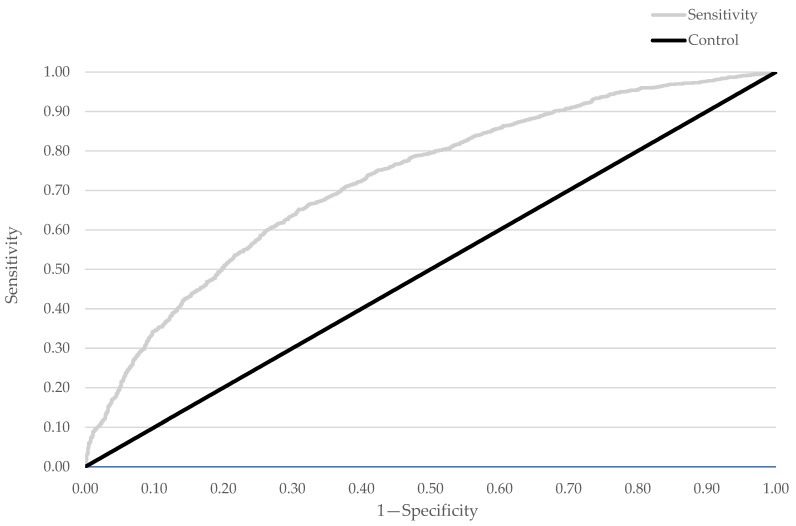
Receiving operating characteristic (ROC) curves for the C-reactive protein predictive model.

From the univariable analysis of various clinical factors, a total of 36 factors were associated with elevated CRP levels. However, only 14 factors remained significant after the multivariable analysis (Table A9). Hydrophilic statin use and higher intensity statin therapy were associated with elevated CRP levels. Other factors independently associated with elevated CRP included residence in non-urban areas; hepatobiliary diseases; higher counts of monocytes, neutrophils and platelets; larger red cell distribution width; higher levels of sodium and alkaline phosphatase; and lower levels of albumin, bilirubin, chloride and renal function. This final multivariable model utilised data from 2604 patients and had a Nagelkerke *R*^2^ value of 0.187. The C-statistic from the ROC analysis was 0.718 (95% CI 0.698–0.738) for the model (Figure 5).

### 3.4. Associations between Elevated CRP and Hospitalisations

**Figure 6:**
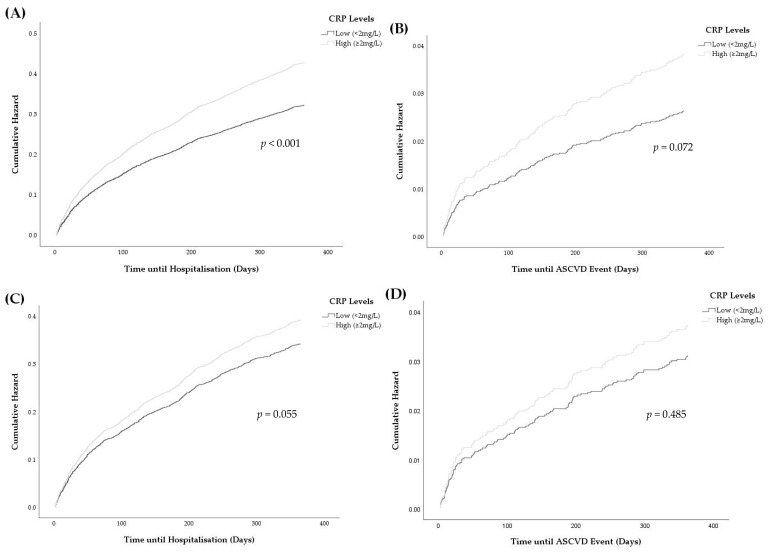
Cumulative risk of all-cause and ASCVD hospitalisations for low (grey) and high (black) CRP levels over the 12-month follow-up period. Model 1 involved adjustments for age and gender, and model 2 involved further adjustments for the independent determinants of CRP. (**A**) Risk of all-cause hospitalisations in model 1. (**B**) Risk of ASCVD hospitalisations in model 1. (**C**) Risk of all-cause hospitalisations in model 2. (**D**) Risk of ASCVD hospitalisations in model 2.

After adjusting for age and gender, elevated CRP was associated with all-cause hospitalisations from 3 months onwards following the CRP measurement (Figure 6A and Table A10). Elevated CRP was not associated with ASCVD hospitalisations in model 1 (Figure 6B), but the cumulative hazard curves for participants with and without elevated CRP levels diverged over time in model 1. These diverging trends of hospitalisation rates were also seen between non-elevated and elevated CRP groups in model 2; however, they were attenuated compared with the respective model 1 curves and were statistically insignificant (Figure 6C,D). Similar statistically insignificant results were obtained after adjustments for BMI and lipid profile in a small sub-cohort of patients with data on BMI and lipid profiles (Table A11).

## 4. Discussion

Comparing the prevalence of lipid-lowering therapy in the elderly between the ePBRN cohort and nationally representative cohorts in the literature provided an insight into longitudinal patterns of ASCVD management [19]. In 2012, the ePBRN cohort had a higher prevalence of lipid-lowering therapy, which was likely due to the higher prevalence of ASCVD risk factors in SWS [10] (41.7% in the ePBRN vs. 34.2% in the general Australian population) [19]. However, in more recent years, the prevalence of lipid-lowering therapy in the general population surpassed that of the ePBRN cohort (40.7% in the ePBRN vs. 44.1% in the general population in 2016) [19]. This suggested that the general population may have begun to present more frequently and receive treatment earlier in the disease progression than the SWS population. Considering that statin prescription rates were stagnant in less urban areas of SWS whilst rising in more urban locations, this may have been related to a lack of healthcare access for residents in more rural areas. Moreover, among subjects taking statins in the ePBRN, the use of low-intensity statin therapy was rare (<3%), indicating that individuals who needed low-intensity statin therapy were either overlooked in health screening protocols or did not present to their GP. Males were over-represented in this subcohort (Table A2), which was consistent with the literature that shows that males present less frequently to the GP than females [20,21]. An exploratory univariable analysis highlighted that males tended to be on using higher-strength statin therapy, indicating that they missed opportunities to be included in early prevention measures and presented with more severe disease (Table A2). This study finding suggested that the health status of the SWS cohort may be declining compared with the general population due to a lack of early intervention, especially in males and non-urban residents.

Lipid-lowering therapy increased in the SWS cohort, rising ~0.5%/year between 2012 and 2019. There was a trend toward hydrophilic statin use, which may be related to the growing favourability of rosuvastatin over atorvastatin in terms of potency, efficacy and safety [22,23,24,25]. Furthermore, despite recent meta-analyses suggesting that statin therapy was effective and safe in the elderly [26], medication usage was significantly lower in those aged ≥85 years compared with other age groups, reflecting prior beliefs that statins provide little benefit to the elderly [15,27]. These trends highlighted the fact that physicians responded to emerging findings in the literature but that it took several years for new evidence to translate into general practice. The timely incorporation of evidence into ASCVD primary care clinical guidelines could be an area for improvement.

Statin use decreased between 2013–2015, which was likely due to a television production aired by the Australian Broadcasting Corporation that was highly critical of statins. This may have caused many patients to refuse statin prescriptions and/or discontinue their use, considering that when the program was withdrawn, statin use rebounded back to prior levels [28]. This exemplified the fact that physicians may encounter barriers due to public opinion, which may be in part shaped by critical media publications such as this production. It reinforced the importance of health advertising in public health, as seen in other campaigns [29]. Interestingly, non-statin lipid-lowering therapy remained steady in the same period, suggesting that compromise and a shift to alternative forms of therapy may be an effective mechanism to address patient concerns, at least temporarily.

Patients on higher-intensity statin therapy were more likely to have elevated CRP. This may indicate that they had worse ASCVD risk profiles due to inadequate control of subclinical inflammation and ASCVD risk [18]. The reason for this inadequate control was likely multifactorial; however, the independent associations of liver function (as assessed by the presence of hepatobiliary conditions, albumin and ALP) and renal function (as assessed by eGFR and electrolytes, such as sodium and chloride) with elevated CRP levels suggested that impaired liver and renal function may exacerbate subclinical inflammation. Therefore, those with other concomitant diseases may be undermanaged relative to typically healthy individuals. More regular screening, improving health literacy, or more aggressively treating ASCVD risk and comorbid conditions may be beneficial in this regard.

Statins are the most widely used lipid-lowering medication but patients often have poor long-term adherence to this therapy [30]. Adherence trend analysis in the ePBRN cohort suggested that statin adherence was 55% in the SWS region compared with the 57% adherence reported in the general population. However, the ePBRN cohort included concession card holders and older individuals, who are generally more compliant with medication [31], whereas the study of the general population excluded these groups. Therefore, the true adherence of the ePBRN cohort may be considerably lower than the general population. Considering that this data reflected prescriptions given to patients, a large barrier to adherence to therapy was presenting to the GP to obtain prescriptions. This was reinforced by the independent association of non-urban residence to lower adherence, which would exacerbate difficulties in making GP visits [32] alongside the other effects that rural residence may have on medication accessibility. Non-urban residence was also independently associated with elevated CRP levels, suggesting that this lower adherence may influence the overall health outcomes. Providing telehealth services may help to circumvent this barrier, as it has been used previously when treating patients with human immunodeficiency virus [33] and when delivering services to rural areas [34].

Elevated CRP was not related to all-cause and ASCVD hospitalisations after accounting for independent determinants of CRP among statin-treated patients. This suggested that monitoring residual ASCVD risk using on-treatment CRP levels would not be effective for statin users in SWS, which is consistent with previous findings from clinical trials [35,36]. However, in the current study, there was a trend in which the cumulative hazard risk of patients with elevated CRP increased relative to those with normal CRP levels over time. A larger sample size with a longer follow-up and more accurate outcome data would aid in drawing a definitive conclusion. Nevertheless, the present study did not support the use of CRP measurement in predicting incident hospitalisation among patients on statin therapy after considering different confounding factors, including demographic factors, statin lipophilicity, total bilirubin and liver enzymes.

There were several limitations to the present study. As EHRs were used as the data source, incomplete/erroneous documentation may reduce the sample size and statistical power of the study and cause misclassifications. Moreover, patients may visit general practices outside the ePBRN network, and thus, a full medical history may not be obtained. There were also significant amounts of missing and outdated data for variables such as smoking status, lipid profiles, BMI and reasons for hospitalisation. This limited the ability to assess the CVD risk profile of subjects taking lipid-lowering medications in the ePBRN data, their underlying reasons for taking these medications and the proportion of these subjects who achieved the lipid target goal. Additionally, the categorisation of medical conditions combined conditions of varying aetiologies. This may cause certain trends and relationships in the dataset to either be overlooked or miscategorised. Furthermore, since pathology laboratory data for different biomarker measurements may not be reported simultaneously, a 2-week window was given to accommodate for this time delay, possibly reducing the accuracy of these biomarker measurements. Moreover, for the analysis of elevated CRP, there was likely oversampling of individuals who had prior indications for pathological tests. Typically, these individuals will be unhealthier and may not have biomarkers that are reflective of the wider populace. Therefore, the findings on elevated CRP and hospitalisation from this study may not be generalisable to the entire SWS population.

Despite these limitations, our study explored the potential use of EHR data to perform a health survey for health surveillance. Prior attempts to establish a population survey in Australia were not successful as few participants presented for blood testing following their interview [37]. However, incorporating a general health survey into already scheduled GP visits may be an effective way to increase turnout and design a viable population survey when focusing on parameters such as CRP.

## 5. Conclusions

Despite the effectiveness of lipid-lowering therapy in clinical trials, there was a lack of monitoring at the population level to ensure this evidence translates into practice. This study reaffirmed the existing health disparity between the SWS population and the Australian general population and suggested that it may be widening due to inadequate identification of individuals with earlier stages of disease. The results suggested that males were presenting with more advanced disease and that needing to visit the GP for a prescription was a barrier to medication adherence. This study also highlighted the need for action at the physician level, such as quicker adoption of new knowledge, involving patients in management plans, and more aggressive management of comorbidities and ASCVD risk factors. Although this study did not support the use of on-treatment CRP levels to monitor the short-term risk of all-cause and ASCVD hospitalisations among statin-treated patients, it demonstrated the potential use of clinical records to monitor population health. Further studies to assess the use of on-treatment CRP levels to monitor the longer-term risk of all-cause and ASCVD hospitalisations in statin-treated patients are warranted.

## Data Availability

The data presented in this study are available upon request from the corresponding author (K.L.O.).

## Appendix A

**Table A1:** Classifications of different lipid-lowering medications based on ATCC codes.

**Medication**	All ATC Codes Searched	ATC Codes Found in ePBRN	Medications Found in ePBRN
**Statins**	C10AA C10BA01-09 C10BA11-12 C10BX	C10AA01 C10AA03 C10AA04 C10AA05 C10AA07 C10BA02 C10BA05 C10BX03	Simvastatin Pravastatin Fluvastatin Atorvastatin Rosuvastatin Simvastatin and ezetimbe Atorvastatin and ezetimbe Atorvastatin and amlodipine
**Fibrates**	C10AB C10BA03 C10BA04 C10BA09 C10BA12	C10AB04 C10AB05	Fenofibrate Gemfibrozil
**Bile acid sequestrants**	C10AC	C10AC01 C10AC02	Cholestyramine Colestipol
**Nicotinic acid**	C10AD C10BA01	C10AD02	Nicotinic acid
**Cholesterol adsorption inhibitors**	C10AX09 C10BA02 C10BA05 C10BA06 C10BA10-12	C10AX09 C10BA02 C10BA05	Ezetimibe Simvastatin and ezetimbe Atorvastatin and ezetimbe
**Other**	C10 (not listed above)	C10AX08	Policosanol

**Table A2:** Classifications of different statins based on intensity and lipophilicity.

Medication Type	Medications
**Statin Intensity**	
** Low**	Simvastatin 10 mg Pravastatin 10–20 mg Fluvastatin 20–40 mg
** Moderate**	Simvastatin 20–40 mg Pravastatin 40–80 mg Fluvastatin 80 mg Atorvastatin 10–20 mg Rosuvastatin 5–10 mg
** High**	Atorvastatin 40–80 mg Rosuvastatin 20–40 mg
**Statin Lipophilicity**	
** Lipophilic**	Simvastatin Fluvastatin Atorvastatin
** Hydrophilic**	Pravastatin Rosuvastatin

**Table A3:** Prescription patterns of lipid-lowering medications in the ePBRN cohort by year.

	2012	2013	2014	2015	2016	2017	2018	2019	*p* for Trend
**Number of total patients**	33,164	35,653	37,968	42,973	50,181	54,432	53,325	46,397	-
**Number of patients on lipid therapy ^**	4689 (14.1)	4914 (13.8)	5213 (13.7)	5630 (13.1)	6903 (13.8)	7795 (14.3)	7819 (14.7)	6824 (14.7)	0.001
**Medication**									
** Statins**	4525 (88.4)	4750 (88.0)	5024 (88.0)	5381 (87.4)	6596 (87.4)	7455 (87.8)	7454 (86.9)	6503 (87.5)	0.009
** Fibrates**	159 (3.1)	185 (3.4)	219 (3.8)	267 (4.3)	331 (4.4)	389 (4.6)	414 (4.8)	373 (5.0)	<0.001
** Bile acid sequestrants**	12 (0.2)	13 (0.2)	16 (0.3)	19 (0.3)	22 (0.3)	28 (0.3)	40 (0.5)	27 (0.4)	0.001
** Nicotinic acid**	2 (0.0)	2 (0.0)	2 (0.0)	6 (0.1)	4 (0.1)	3 (0.0)	4 (0.0)	0 (0.0)	0.332
** Cholesterol adsorption Inhibitors**	419 (8.2)	448 (8.3)	447 (7.8)	482 (7.8)	594 (7.9)	618 (7.3)	670 (7.8)	529 (7.1)	0.008
** Other**	0 (0.0)	0 (0.0)	0 (0.0)	0 (0.0)	1 (0.0)	0 (0.0)	0 (0.0)	0 (0.0)	0.959
**Form of Therapy**									
** Monotherapy (single medication)**	4276 (91.6)	4451 (91.0)	4741 (91.3)	5131 (91.5)	6298 (91.5)	7131 (91.8)	7100 (91.1)	6247 (91.8)	0.493
** Combination therapy (2 or more)**	413 (8.4)	463 (9.0)	472 (8.7)	499 (8.5)	605 (8.5)	664 (8.2)	719 (8.9)	577 (8.2)	0.493
**Statin Intensity ***									
** Low**	103 (2.5)	107 (2.49)	110 (2.41)	114 (2.33)	157 (2.61)	166 (2.44)	164 (2.43)	133 (2.24)	0.518
** Moderate**	2765 (67.1)	2856 (66.5)	3040 (66.6)	3266 (66.7)	4020 (66.9)	4581 (67.4)	4506 (66.7)	3947 (66.5)	0.913
** High**	1250 (30.4)	1332 (31.0)	1413 (31.0)	1516 (31.0)	1831 (30.5)	2055 (30.2)	2082 (30.8)	1857 (31.3)	0.743
**Statin Lipophilicity ***									
** Lipophilic**	2415 (58.6)	2407 (56.0)	2425 (53.1)	2451 (50.1)	2905 (48.4)	3105 (45.6)	3092 (45.8)	2704 (45.5)	<0.001
** Hydrophilic**	1705 (41.4)	1891 (44.0)	2139 (46.9)	2445 (49.9)	3103 (51.6)	3701 (54.4)	3662 (54.2)	3233 (54.5)	<0.001

Data are shown as *n* (%). * Statin intensity was assessed in any individual taking statins, both as a monotherapy and combination therapy. ^ This value may be lower than the sum of the values underneath, as some patients were on a combination therapy, which was treated as separate records in the prevalence analysis.

**Table A4:** Analyses of statin intensity by gender.

	Male	Female	Odds Ratio for Female Gender (95% CI)	*p*-Value
Low-intensity statin	33 (37.9%)	54 (62.1%)	Referent	-
Moderate-intensity statin	926 (44.0%)	1177 (56.0%)	0.78 (0.50–1.21)	0.262
High-intensity statin	558 (56.6%)	428 (43.4%)	0.47 (0.30–0.74)	0.001

**Table A5:** Demographics of patients taking lipid-lowering medications at different timepoints in the ePBRN cohort.

	2012	2013	2014	2015	2016	2017	2018	2019	*p* for Trend
***n***	4689	4914	5213	5630	6903	7795	7819	6824	0.001
**Age (years)**	64.0 (12.4)	64.6 (12.4)	64.9 (12.2)	65.0 (12.2)	65.3 (12.1)	65.2 (12.3)	65.5 (12.2)	66.5 (11.9)	<0.001
**Males (%)**	51.4	51.8	51.6	52.6	51.8	50.8	51.7	51.5	0.596
**ATSI (%)**	1.2	1.2	1.1	1.1	1.1	1.3	1.3	1.5	0.121
**Living location (%)**									
** Urban**	52.6	54.1	53.9	53.6	57.7	58.5	58.0	57.8	
** Non-urban**	47.4	45.9	46.1	46.4	42.3	41.5	42.0	42.2	<0.001

Data are shown as *n* (%) or mean (SD). The p for the time trend was estimated using linear regression or logistic regression where appropriate. Some values may not add to 100% due to rounding. Abbreviation: ATSI—Aboriginals and Torres Strait Islanders.

**Table A6:** Prevalence patterns within demographic subgroups.

	2012	2013	2014	2015	2016	2017	2018	2019	*p* for Trend
Age Group									
Any Lipid Therapy									
18–44	335 (1.9)	304 (1.6)	302 (1.5)	321 (1.5)	356 (1.4)	438 (1.5)	405 (1.5)	286 (1.3)	<0.001
45–54	779 (12.2)	788 (11.8)	787 (11.3)	835 (11.2)	994 (10.6)	1127 (12.3)	1083 (12.2)	860 (11.2)	0.797
55–64	1433 (30.9)	1455 (28.7)	1557 (27.8)	1652 (25.3)	1978 (26.4)	2225 (27.7)	2217 (28.0)	1873 (25.9)	<0.001
65–74	1176 (43.9)	1325 (44.5)	1449 (43.7)	1615 (39.9)	2050 (44.4)	2321 (45.3)	2379 (45.5)	2174 (43.0)	0.295
75–84	784 (49.9)	828 (47.9)	894 (48.0)	955 (43.5)	1202 (49.0)	1324 (50.5)	1355 (51.7)	1289 (51.4)	<0.001
85+	182 (39.5)	214 (40.4)	224 (37.0)	252 (31.0)	323 (35.6)	360 (35.7)	380 (37.8)	342 (35.6)	0.307
Statin Therapy									
18–44	310 (1.8)	287 (1.5)	281 (1.4)	293 (1.3)	328 (1.2)	399 (1.4)	373 (1.4)	266 (1.2)	<0.001
45–54	753 (11.8)	766 (11.4)	755 (10.9)	794 (10.6)	936 (10.0)	1072 (11.7)	1017 (11.4)	809 (10.5)	0.797
55–64	1381 (29.8)	1407 (27.7)	1499 (26.8)	1596 (24.4)	1903 (25.4)	2144 (26.7)	2127 (26.9)	1797 (24.9)	<0.001
65–74	1140 (42.6)	1279 (43.0)	1401 (42.3)	1541 (38.1)	1970 (42.6)	2216 (43.3)	2270 (43.4)	2062 (40.7)	0.966
75–84	764 (48.7)	800 (46.3)	869 (46.7)	913 (41.6)	1144 (46.6)	1273 (48.6)	1299 (49.6)	1236 (49.2)	0.005
85+	177 (38.4)	211 (39.8)	219 (36.1)	244 (30.1)	315 (34.7)	351 (34.8)	368 (36.6)	333 (34.7)	0.256
Non-Statin Therapy									
18–44	25 (0.1)	17 (0.1)	21 (0.1)	28 (0.1)	28 (0.1)	39 (0.1)	32 (0.1)	20 (0.1)	0.557
45–54	26 (0.4)	22 (0.3)	32 (0.5)	41 (0.6)	58 (0.6)	55 (0.6)	66 (0.8)	51 (0.7)	<0.001
55–64	52 (1.1)	48 (1.0)	58 (1.0)	56 (0.9)	75 (1.0)	81 (1.0)	90 (1.1)	76 (1.1)	0.614
65–74	36 (1.4)	46 (1.6)	48 (1.5)	74 (1.8)	80 (1.7)	105 (2.1)	109 (2.1)	112 (2.2)	<0.001
75–84	20 (1.3)	28 (1.6)	25 (1.3)	42 (1.9)	58 (2.4)	51 (2.0)	56 (2.1)	53 (2.1)	0.010
85+	5 (1.1)	3 (0.6)	5 (0.8)	8 (1.0)	8 (0.9)	9 (0.9)	12 (1.2)	9 (0.9)	<0.001
Sex Group									
Any Lipid Therapy									
Male	2411 (16.4)	2544 (15.9)	2688 (15.8)	2960 (15.5)	3566 (15.9)	3952 (16.2)	4036 (17.0)	3513 (17.1)	<0.001
Female	2277 (12.4)	2369 (12.1)	2523 (12.1)	2668 (11.2)	3335 (12.0)	3839 (12.8)	3780 (12.9)	3309 (12.9)	<0.001
Statin Therapy									
Male	2324 (15.8)	2464 (15.4)	2585 (15.2)	2830 (14.8)	3413 (15.2)	3779 (15.5)	3843 (16.2)	3349 (16.3)	0.002
Female	2200 (12.0)	2285 (11.6)	2437 (11.7)	2549 (10.7)	3181 (11.5)	3672 (12.3)	3608 (12.3)	3152 (12.3)	0.001
Non-Statin Therapy									
Male	87 (0.59)	80 (0.5)	103 (0.61)	130 (0.68)	153 (0.69)	173 (0.71)	193 (0.81)	164 (0.8)	<0.001
Female	77 (0.42)	84 (0.43)	86 (0.41)	119 (0.5)	154 (0.55)	167 (0.55)	172 (0.59)	157 (0.61)	<0.001
Living Location									
Any Lipid Therapy									
Urban	2460 (11.3)	2652 (11.2)	2807 (11.2)	3016 (10.4)	3972 (12.1)	4552 (13.1)	4527 (13.9)	3940 (14.1)	<0.001
Non-urban	2220 (19.7)	2253 (19.1)	2400 (18.7)	2606 (19.0)	2912 (17.0)	3228 (16.6)	3283 (16.0)	2877 (15.8)	<0.001
Statin Therapy									
Urban	2358 (10.8)	2549 (10.8)	2689 (10.8)	2868 (9.8)	3783 (11.5)	4347 (12.5)	4326 (13.3)	3755 (13.4)	<0.001
Non-urban	2158 (19.1)	2192 (18.6)	2329 (18.1)	2505 (18.3)	2795 (16.3)	3093 (15.9)	3119 (15.2)	2741 (15.0)	<0.001
Non-Statin Therapy									
Urban	102 (0.47)	103 (0.43)	118 (0.47)	148 (0.51)	189 (0.58)	205 (0.59)	201 (0.61)	185 (0.66)	<0.001
Non-urban	62 (0.55)	61 (0.52)	71 (0.55)	101 (0.74)	117 (0.68)	135 (0.69)	164 (0.8)	136 (0.75)	<0.001

**Table A7:** Univariable and multivariable analyses of demographic factors associated with lipid-lowering therapy adherence.

Demographic Factor	*n*	Univariable Analysis		Multivariable Analysis	
		OR (95% CI)	*p*	OR (95% CI)	*p*
Age (year)	9109	1.03 (1.02–1.03)	<0.001	1.03 (1.02–1.03)	<0.001
Male gender	4670	0.99 (0.91–1.08)	0.846	-	-
ATSI	117	0.70 (0.48–1.00)	0.052	0.74 (0.51–1.07)	0.108
Non-urban residence	3937	0.79 (0.73–0.86)	<0.001	0.78 (0.71–0.85)	<0.001

Abbreviations: ATSI—Aboriginals and Torres Strait Islanders; CI—confidence interval; OR—odds ratio.

**Table A8:** Baseline characteristics of the participants from the ePBRN cohort.

Parameter	*n*	Estimate	Missing Data (%)
Demographic and Behavioural Factors			
Age	3224	67.2 (12.9)	0 (0.0)
Male gender	1543	47.9	0 (0.0)
ATSI	24	0.8	28 (0.9)
Smoking status			
Non-smoker	1363	48.9	
Ex-smoker	1108	39.7	
Smoker	319	11.4	
Overall	2790		434 (13.5)
Non-urban residence	1118	34.7	5 (0.2)
Number of visits to GP in past year	2918	19.6 (13.8)	306 (9.5)
Elevated CRP	1842	57.1	0 (0.0)
Medical Conditions			
Arthritis	1412	43.8	0 (0.0)
Cancer	443	13.7	0 (0.0)
Atherosclerotic cardiovascular Disease	975	30.2	0 (0.0)
Other cardiac disease	638	19.8	0 (0.0)
Other vascular disease	174	5.4	0 (0.0)
Diabetes	1144	35.5	0 (0.0)
Dyslipidaemia	997	30.9	0 (0.0)
Gastrointestinal	834	25.9	0 (0.0)
Hepatobiliary	193	6.0	0 (0.0)
Hypertension	1945	60.3	0 (0.0)
Neurological	140	4.3	0 (0.0)
Psychiatric	809	25.1	0 (0.0)
Renal	241	7.5	0 (0.0)
Respiratory	810	25.1	0 (0.0)
Medication History			
Anti-thrombotic medication	1253	38.9	0 (0.0)
Diabetic medication ^a^	915	28.4	0 (0.0)
Cardiovascular medication	2370	73.5	0 (0.0)
Statin intensity			
Low	87	2.7	
Moderate	2103	66.2	
High	986	31.1	
Overall	3176		48 (1.5)
Lipophilic statin	1575	48.9	0 (0.0)
Clinical Characteristics			
Examination Results			
BMI (kg/m^2^)	2266	29.5 (26.0–33.6)	958 (29.7)
Systolic blood pressure (mmHg) ^b^	888	132.33 (16.7)	2336 (72.5)
Diastolic blood pressure (mmHg) ^b^	888	77.10 (10.5)	2336 (72.5)
Full Blood Count			
Haemoglobin (g/L)	3117	135.6 (17.8)	107 (3.3)
Mean red cell haemoglobin (pg)	3085	29.8 (2.3)	139 (4.3)
Mean red cell haemoglobin Concentration (g/L)	3085	330.3 (13.1)	139 (4.3)
Mean red cell volume (fL)	3117	90.4 (6.0)	107 (3.3)
RCC (10^12^/L) ^a^	3113	4.6 (0.6)	111 (3.4)
RDW (%)	3050	13.1 (12.3–14.0)	174 (5.4)
Monocyte (10^9^/L)	3115	0.6 (0.5–0.7)	109 (3.4)
Neutrophil (10^9^/L)	3117	4.5 (3.5–6.0)	107 (3.3)
Lymphocyte (10^9^/L)	3117	2.0 (1.5–2.6)	107 (3.3)
WBC (10^9^/L) ^a^	3118	7.6 (6.2–9.2)	106 (3.3)
Platelet (10^9^/L)	3113	243.7 (75.0)	111 (3.4)
Serum Biochemistry			
Bicarbonate (mmol/L)	3029	25.9 (3.3)	195 (6.1)
Chloride (mmol/L)	3032	102.5 (4.3)	192 (6.0)
Potassium (mmol/L)	2978	4.5 (0.5)	246 (7.6)
Sodium (mmol/L)	3032	140.0 (3.2)	192 (6.0)
Liver Function Tests			
Albumin (g/L)	2977	41.9 (4.12)	247 (7.7)
Bilirubin (µmol/L)	2843	9.0 (6–12)	381 (11.8)
ALT (U/L)	2847	23 (17–33)	377 (11.7)
AST (U/L)	2701	23 (20–30)	523 (16.2)
ALP (U/L)	2853	75 (61–92)	371 (11.5)
GGT (U/L)	2852	30 (19–51)	372 (11.5)
Total protein (g/L)	2856	70.6 (5.9)	368 (11.4)
Kidney Function Tests			
Creatinine (µmol/L)	3031	80 (67–96)	193 (6.0)
eGFR			
1	507	17.6	
2	811	28.2	
3a	296	10.3	
3b	344	12.0	
4	634	22.0	
5	286	9.9	
Overall	2878		346 (10.7)
Urea (mmol/L)	3029	6.4 (5.1–8.1)	195 (6.1)
Lipid Profiles			
Total cholesterol (mmol/L) ^b^	1242	4.5 (1.2)	1982 (61.5)
HDL-C (mmol/L) ^b^	942	1.3 (0.4)	2282 (70.8)
LDL-C (mmol/L) ^b^	902	2.4 (1.0)	2322 (72.0)
Non-HDL cholesterol (mmol/L) ^b^	942	3.2 (1.1)	2282 (70.8)
Total:HDL cholesterol ratio^b^	942	3.6 (1.2)	2282 (70.8)
Triglyceride (mmol/L) ^b^	1236	1.4 (1.0–2.1)	1988 (61.7)
Blood Glucose			
Glucose (fasting) (mmol/L) ^b^	550	5.5 (4.9–6.5)	2674 (82.9)
Glucose (random) (mmol/L) ^b^	1876	6.2 (5.3–7.9)	1348 (41.8)
HbA1c (%) ^b^	1142	6.3 (5.7–7.4)	2082 (64.6)

Data are shown as a percentage, mean (SD) or median (IQR) where appropriate. Abbreviations: ALP—alkaline phosphatase; ALT—alanine aminotransferase; AST—aspartate aminotransferase; ATSI—Aboriginals and Torres Strait Islanders; CI—confidence interval; eGFR—estimated glomerular filtration rate; GGT—γ-glutamyl transferase; HbA1c—glycosylated haemoglobin; HDL-C—high-density lipoprotein cholesterol; LDL-C—low-density lipoprotein cholesterol; MCH—mean cell haemoglobin; MCHC—mean cell haemoglobin concentration; MCV—mean cell volume; ns—not significant after backward elimination; OR—odds ratio; RCC—red cell count; RDW—red cell distribution width; WBC—white blood cell count. ^a^ Excluded from the multivariable analysis due to high multicollinearity with other variables present. ^b^ Incorporated into other variables.

**Table A9:** Association of on-treatment C-reactive protein levels with patient factors in the ePBRN cohort.

Parameters		Univariable Analysis		Multivariable Analysis	
	*n*	OR (95% CI)	*p*	OR (95% CI)	*p*
Demographic and Behavioural Factors					
Age	3224	1.01 (1.00–1.01)	0.032	ns	ns
Male gender	1543	0.94 (0.82–1.08)	0.370	-	-
ATSI	24	2.25 (0.89–5.67)	0.087	ns	ns
Smoking status					
Non-smoker	1363	Referent	-	-	-
Ex-smoker	1108	1.24 (1.06–1.46)	0.008	ns	ns
Smoker	319	1.41 (1.10–1.81)	0.007	ns	ns
Overall			0.012	ns	ns
Non-urban residence	1118	1.92 (1.65–2.24)	<0.001	1.76 (1.46–2.13)	<0.001
Number of visits to GP in past year	2918	1.01 (1.01–1.02)	<0.001	ns	ns
Medical Conditions					
Arthritis	1412	1.02 (0.88–1.17)	0.815	-	-
Cancer	443	1.04 (0.85–1.28)	0.687	-	-
Atherosclerotic cardiovascular Disease	975	0.97 (0.83–1.13)	0.695	-	-
Other cardiac disease	638	1.17 (0.98–1.40)	0.082	ns	ns
Other vascular disease	174	1.04 (0.76–1.42)	0.803	-	-
Diabetes	1144	1.13 (0.98–1.32)	0.100	ns	ns
Dyslipidaemia	997	0.83 (0.72–0.96)	0.012	ns	ns
Gastrointestinal	834	1.05 (0.90–1.23)	0.542	-	-
Hepatobiliary	193	1.55 (1.14–2.11)	0.005	1.71 (1.16–2.52)	0.007
Hypertension	1945	1.03 (0.89–1.18)	0.724	-	-
Neurological	140	0.74 (0.53–1.04)	0.082	-	-
Psychiatric	809	1.06 (0.90–1.25)	0.471	-	-
Renal	241	1.41 (1.07–1.86)	0.014	ns	ns
Respiratory	810	1.18 (1.0–1.39)	0.047	ns	ns
Medication History					
Anti-thrombotic medication	1253	1.16 (1.0–1.33)	0.048	ns	ns
Diabetic medication ^a^	915	1.09 (0.93–1.27)	0.297	-	-
Cardiovascular medication	2370	1.30 (1.11–1.52)	0.001	ns	ns
Statin intensity					
Low	87	Referent	-	-	-
Moderate	2103	1.43 (0.93–2.20)	0.100	1.42 (0.85–2.37)	0.176
High	986	1.24 (0.80–1.93)	0.329	1.14 (0.68–1.91)	0.629
Overall			0.067		0.032
Hydrophilic statin	1575	1.09 (0.95–1.26)	0.204	1.27 (1.07–1.51)	0.007
Clinical Characteristics					
Examination Results					
BMI (kg/m^2^)	2266	1.02 (1.01–1.04)	<0.001	-	-
Systolic blood pressure (mmHg) ^b^	888	1.00 (0.99–1.01)	0.902	-	-
Diastolic blood pressure (mmHg) ^b^	888	1.00 (0.99–1.01)	0.810	-	-
Full Blood Count					
Haemoglobin (g/L)	3117	0.99 (0.99–0.99)	<0.001	ns	ns
MCH (pg)	3085	0.95 (0.92–0.98)	0.001	ns	ns
MCHC (g/L)	3085	0.99 (0.98–0.99)	<0.001	ns	ns
MCV (fL)	3117	0.99 (0.98–1.00)	0.209	-	-
RCC (10^12^/L) ^a^	3113	0.87 (0.78–0.98)	0.019	-	-
RDW (%)	3050	1.21 (1.15–1.27)	<0.001	1.20 (1.13–1.28)	<0.001
Monocyte (10^9^/L)	3115	2.77 (2.02–3.78)	<0.001	2.64 (1.72–4.04)	<0.001
Neutrophil (10^9^/L)	3117	1.17 (1.13–1.21)	<0.001	1.09 (1.04–1.13)	<0.001
Lymphocyte (10^9^/L)	3117	0.99 (0.96–1.02)	0.566	-	-
WBC (10^9^/L) ^a^	3118	1.11 (1.08–1.14)	<0.001	ns	ns
Platelet (10^9^/L)	3113	1.00 (1.00–1.01)	<0.001	1.00 (1.00–1.00)	<0.001
Serum Biochemistry					
Bicarbonate (mmol/L)	3029	1.01 (0.99–1.03)	0.358	-	-
Chloride (mmol/L)	3032	0.96 (0.94–0.98)	<0.001	0.92 (0.90–0.95)	<0.001
Potassium (mmol/L)	2978	0.95 (0.82–1.10)	0.475	-	-
Sodium (mmol/L)	3032	0.98 (0.96–1.01)	0.195	1.07 (1.03–1.11)	0.001
Liver Function Tests					
Albumin (g/L)	2977	0.95 (0.93–0.97)	<0.001	0.96 (0.94–0.99)	0.001
Bilirubin (µmol/L)	2843	0.98 (0.97–1.00)	0.005	0.98 (0.97–1.00)	0.007
ALT (U/L)	2847	1.00 (1.00–1.01)	0.052	ns	ns
AST (U/L)	2701	1.00 (1.00–1.00)	0.101	ns	ns
ALP (U/L)	2853	1.02 (1.01–1.02)	<0.001	1.01 (1.01–1.02)	<0.001
GGT (U/L)	2852	1.00 (1.00–1.00)	<0.001	ns	ns
Total protein (g/L)	2856	0.99 (0.98–1.01)	0.415	-	-
Kidney Function Tests					
Creatinine (µmol/L)	3031	1.00 (1.00–1.00)	0.009	ns	ns
eGFR					
1	507	1.01 (0.75–1.35)	0.971	0.89 (0.64–1.24)	0.493
2	811	1.06 (0.81–1.39)	0.670	1.20 (0.82–1.74)	0.349
3a	296	1.64 (1.17–2.29)	0.004	1.54 (1.03–2.30)	0.035
3b	344	1.29 (0.94–1.77)	0.119	1.51 (1.07–2.12)	0.019
4	634	1.08 (0.82–1.43)	0.593	1.40 (0.97–2.01)	0.071
5	286	Referent	-	Referent	
Overall	2878		0.014		0.003
Urea (mmol/L)	3029	1.04 (1.02–1.06)	<0.001	ns	ns
Lipid Profiles					
Total cholesterol (mmol/L) ^b^	1242	1.08 (0.98–1.19)	0.125	-	-
HDL-C (mmol/L) ^b^	942	0.74 (0.53–1.05)	0.095	-	-
LDL-C (mmol/L) ^b^	902	1.18 (1.02–1.35)	0.023	-	-
Non-HDL cholesterol (mmol/L) ^b^	942	1.16 (1.02–1.30)	0.019	-	-
Total:HDL-C ratio ^b^	942	1.21 (1.08–1.36)	0.001	-	-
Triglyceride (mmol/L) ^b^	1236	1.06 (0.97–1.16)	0.201	-	-
Blood Glucose					
Glucose (fasting) (mmol/L) ^b^	550	1.03 (0.97–1.09)	0.387	-	-
Glucose (random) (mmol/L) ^b^	1876	1.04 (1.01–1.07)	0.011	-	-
HbA1c (%) ^b^	1142	1.14 (1.06–1.23)	0.001	-	-

Abbreviations: ALP—alkaline phosphatase; ALT—alanine aminotransferase; AST—aspartate aminotransferase; ATSI—Aboriginals and Torres Strait Islanders; CI—confidence interval; eGFR—estimated glomerular filtration rate; GGT—γ-glutamyl transferase; HbA1c—glycosylated haemoglobin; HDL-C—high-density lipoprotein cholesterol; LDL-C—low-density lipoprotein cholesterol; MCH—mean cell haemoglobin; MCHC—mean cell haemoglobin concentration; MCV—mean cell volume; ns—not significant after backward elimination; OR—odds ratio; RCC—red cell count; RDW—red cell distribution width; WBC—white blood cell count. ^a^ Excluded from the multivariable analysis due to high multicollinearity with other variables present. ^b^ Incorporated into other variables.

**Table A10:** Association of elevated CRP levels (≥2 mg/L) with different hospitalisation events.

Events	Model 1 (*n* = 3224)		Model 2 (*n* = 2562)	
	Event Rate (%)	Elevated CRP OR (95% CI)	Event Rate (%)	Elevated CRP OR (95% CI)
All Hospitalisations				
1 month	287	1.15 (0.91–1.46)	248 (7.7)	0.99 (0.75–1.29)
3 months	546	1.30 (1.09–1.55) *	441 (13.7)	1.06 (0.87–1.31)
6 months	773	1.29 (1.11–1.49) *	611 (19.0)	1.10 (0.92–1.31)
12 months	1077	1.32 (1.17–1.50) *	861 (26.7)	1.16 (1.00–1.34)
ASCVD Hospitalisations				
1 month	36	0.58 (0.3–1.12)	35 (1.1)	0.57 (0.30–1.16)
3 months	54	0.91 (0.53–1.55)	52 (1.6)	0.82 (0.46–1.46)
6 months	83	1.15 (0.74–1.79)	77 (2.4)	1.02 (0.62–1.65)
12 months	131	1.39 (0.97–2.00)	117 (3.6)	1.16 (0.77–1.73)

* *p* < 0.05. Abbreviations: ASCVD—atherosclerotic cardiovascular disease; CI—confidence interval; OR—odds ratio. Model 1: adjusted for age and gender. Model 2: model 1 further adjusted for non-urban residence, hepatobiliary conditions, statin intensity, statin lipophilicity, albumin, ALP, bilirubin, chloride, eGFR, monocytes, neutrophils, platelets, RDW and sodium.

**Table A11:** Association of elevated CRP levels (≥2 mg/L) with different hospitalisation events at 12 months with adjustment for BMI and lipid parameters in a sub-cohort.

Events	Model 3 (*n* = 1171)		Model 4 (*n* = 414)	
	Event Rate (%)	CRP ≥2 mg/L OR (95% CI)	Event Rate (%)	CRP ≥ 2 mg/L OR (95% CI)
All-cause hospitalisations	399 (34.1)	1.13 (0.91–1.40)	99 (23.9)	0.99 (0.62–1.58)
ASCVD hospitalisations	62 (2.3)	1.13 (0.66–1.96)	22 (5.3)	1.54 (0.56–4.25)

Abbreviations: ASCVD—atherosclerotic cardiovascular disease; CI— confidence interval; OR—odds ratio. Model 3: model 2 from Table A8 with a further adjustment for BMI. Model 4: model 3 with further adjustments for total cholesterol, HDL cholesterol and triglycerides.
